# Unversity Clinique

**Published:** 1854-03

**Authors:** S. D. Gross

**Affiliations:** Professor of Surgery in the University of Louisville


### MEDICINE AND SURGERY.

MARCH, 1854. **NEW SERIES.** VOL. I.—NO. 3.

### ART. I.—UNVERSITY CLINIQUE.—CASES AND OBSERVATIONS IN SURGERY.

BY S. D. GROSS, M. D., PROFESSOR OF SURGERY IN THE UNIVERSITY OF LOUISVILLE.

CASE 1.—*Scrotal Hernia.—A Fact in Legal Medicine.*

THE present case is one of scrotal hernia, the tumor, as you perceive, being of large bulk, and, as I learn, of fourteen years duration. The patient, whose name is Marsahll, is an Irishman, aged 74 years, of intemperate habits, and a shoemaker by occupation. The disease, it appears, came on without any assignable cause. The protrusion, which exists on the left side, has never occasioned him any pain, and he has, therefore, permitted it to pursue its own course, having never worn a truss or suspensory bandage. It is impossible to determine, from the history of the case, whether the parts originally passed through the internal ring, or whether the rupture was one by the direct descent, as it is called. The tumor, at present, is twelve inches in length, and nearly two feet in circumference, at its widest part, which is at the middle; its figure, as you perceive, being somewhat pyriform. It is firm, but somewhat elastic to the touch, and can be handled rather roughly without giving pain. The testicle is situated at its inferior extremity, and the head of the penis is nearly buried in its substance above. From the fact that the man has incontinence of urine, which constantly dribbles off, he is very uncomfortable, and not a little offensive. He frequently experiences dragging sensations and colicky pains in the abdomen, and the bowels are habitually constipated. The contents of the tumor are, in a great measure, irreducible.

To render the case of Marshall instructive to you, it is necessary that I should offer some remarks upon the nature of the disease with which he is afflicted. We have just seen that the tumor, which is an unusually large one, is irreducible. The question, then, arises, how has it become irreducible? Upon this subject the patient is unable to afford us any intelligence, and hence we are necessarily left to our own conjectures respecting it. If we could cut down upon the parts, and examine them as they lie in their prison-chamber, we could very easily determine the question; but such a procedure would be unjustifiable, and is not for a moment to be entertained. Experience has shown that various circumstances may render a hernia, originally reducible, ultimately irreducible. Among these, one of the most common is the formation of adhesions between the protruded parts and the inner surface of the sac, produced by effused lymph. Sometimes bands of this substance are extended across the protruded parts, or from the protruded parts to the sides of the sac, thus tying them to each other, and rendering their replacement impracticable. Occasionally the irreducibility depends upon the abnormal enlargement of the parts, induced by their long residence in their unnatural and extra-abdominal situation. Both bowel and omentum may undergo this change, but it is well known to be much more frequent, as well as much more extensive, in the latter than in the former. Again, the cause may reside in the ring, or aperture of descent, which may be rendered disproportionably small; or the protruded viscera may contract adhesions to the edges of the opening, and in this manner they may be prevented from re-ascending into their original situation. Finally, the surgeon may be unable to effect a reduction on account of the long sojourn of the hernial contents on the outside of the peritoneal cavity, and the consequent diminution of the size of this cavity, thereby destroying its capacity for the reception and accommodation of the dislocated viscera. Whether the latter cause has any agency in preventing a reduction in Marshall's case, we cannot positively assert; but it is reasonable to suppose that it has, especially when we take into consideration the size and age of the tumor.

We must inquire, in the next place, into the nature of the tumor. I have stated that the man has scrotal hernia, but I have not yet given you any reasons for my opinion, and, unless this be done, the chief interest of the case will be lost to you. From your knowledge of scrotal hernia, superficial as it may be, you are aware that this disease is liable to be confounded with other affections, particularly with hydrocele, variocele, and sarcocele. In a case like the present, where the symptoms are so well marked, mistake is hardly possible; but you must not expect always to have the disease spread out before you in so clear and tangible a form.

The disease with which such a tumor as this is most liable to be confounded is hydrocele. From this, however, it may always be distinguished by bearing in mind the following circumstances: Hernia always begins above, showing itself, in the first instance, as a tumor in the groin, from which it gradually descends into the scrotum. In hydrocele the reverse is the case, the swelling commencing below, and gradually extending upwards. In hernia the tumor is irregular in shape, generally more or less flattened in front and behind; whereas in hydrocele it is usually pyriform, being larger below than above. In hernia the testicle is at the bottom of the tumor; while in hydrocele it is at its posterior surface, commonly above the junction of the inferior with the two superior thirds, though this arrangement, as I remarked to you on a former occasion, is by no means constant; for in many cases of hydrocele the organ lies at the base of the swelling. In hernia the tumor is doughy, or gaseous, not elastic and fluctuating as in hydrocele; opake, and not translucent; in the former the patient usually experiences disagreeable dragging sensations and colicky pains, especially when the protrusion is very large; while in the latter he suffers no inconvenience save what results from the volume and weight of the swelling. In reducible hernia the contents of the tumor are easily replaced when the patient is recumbent, but re-descend the moment he resumes the erect posture; while in hydrocele no such changes can possibly occur, whatever may be the posture. When we add to these symptoms the fact that the spermatic cord is always behind the protruded parts in hernia, and, consequently, much less distinct in its outline than in hydrocele, in which it can almost always be felt as a firm, rounded body at the upper extremity of the tumor, and the circumstance that the opening through which the rupture has taken place can always be satisfactorily traced with the finger, while in hydrocele the inguinal rings retain their natural form; we shall have no difficulty in arriving, in the majority of instances, at a correct decision. Much valuable information may also be derived from the history of the case, and from the use of the exploring needle, which, whenever there is any doubt about the matter, will not fail to afford the requisite light.

Scrotal hernia can always be readily distinguished from varicocele by the peculiar feel which the enlarged veins in this disease impart to the finger, which is similar to that of a bundle of earthworms, or of the intestines of a squirrel; by the bluish appearance of the tumor; and by the circumstance that the swelling, after being effaced, is always promptly reproduced when the patient is placed erect, and pressure is applied to the external abdominal ring. In reducible hernia, on the contrary, such a procedure necessarily prevents the re-formation of the tumor. In hernia, moreover, the swelling receives a distinct impulse under coughing and other muscular exertion; while in variocele the parts are perfectly passive.

In sarcocele the best guides are the history of the case, the uniform hardness of the swelling, the normal state of the abdominal rings, the inability of the surgeon to affect the volume of the tumor by his manipulations, and the indurated and distended condition of the scrotum. When the disease is associated with hydrocele, a part of the tumor will be likely to be translucent, soft, and fluctuating; thus strikingly contrasting with the remainder.

Solid tumors—fibrous, adipous, sebaceous, cystic, and encephaloid—developed in the scrotum, testicle, or vaginal tunic, are, in general, easily distinguished by their progress, by their form and consistence, by the nature of the local distress, and by the presence or absence of constitutional involvement.

The next question we are to consider in regard to this disease is the *treatment.* This, in the case before us, resolves itself into very simple means. If the affection were recent, an attempt might be made to reduce the parts, or, rather, to render them reducible, but the time for this has passed by, and we must, therefore, content ourselves with palliatives. For this purpose the tumor should be constantly supported, both day and night, with a suspensory bandage, provided with shoulder-straps, otherwise it will answer the purpose but indifferently. By means of such an apparatus the patient will be relieved of much of his inconvenience, at the same time that the protrusion will be pretty effectually guarded against further increase. Great attention should also be paid to the bowels, which should be constantly maintained in a soluble condition. The diet should be plain and simple, easy of digestion, and comprised in the smallest possible bulk, lest the alimentary tube should suffer from flatulence and fœcal distention. All violent bodily exertion, fatiguing walks, and exercise on horseback must be carefully avoided. Such, gentlemen, in a few words, are the means to which we are restricted in the case of Marshall. I have said nothing about the treatment of his incontinence of urine. For the relief of this, I shall order for him, three times a day, thirty grains of bicarbonate of soda, dissolved in a third of a tumblerful of cistern water, and taken about two hours after each meal. The alkali may, perhaps, assist in allaying the morbid sensibility of the mucous membrane of the neck of the bladder, by correcting the acrimony of the urine, and thus rendering the organ more tolerant of its contents. Generally, however, but little benefit results from any mode of treatment that can be adopted, and the poor patient is obliged to bear with his infirmity the remainder of his days. Such an event is particularly to be looked for when the disease depends, as it often does, under such circumstances, upon paralysis of the bladder, and not upon mere irritability of the mucous membrane.

If Marshall's case were one of recent standing, an attempt might be made, and not without some prospect of success, to render the hernia reducible, by removing the adhesions which have led to the incarceration of its contents. The treatment necessary for fulfilling this indication would consist, first, in the observance of the most thorough and persistent recumbency; secondly, in the use of the smallest possible quantity of food consistent with the preservation of life; thirdly, in the steady and careful suspension of the parts; fourthly, in the application of sorbefacients; and, finally, in the exhibition of mercurials with a view to the production and maintenance of slight ptyalism. When the patient is robust and plethoric, general bleeding and active purgation are premised; but in ordinary cases these remedies are uncalled for. Under this treatment, carefully persisted in for several months, the morbid adhesions are occasionally destroyed, and the parts rendered reducible. Little confidence, however, it must be confessed, is to be placed in it, and in the few instances in which I have tried it it has utterly failed.

In connection with the case of Marshall, I may mention another, of a similar nature, which has lately occurred in this city, and which has been a source of much interest in a medico-legal point of view. I allude to the case of an old gentleman of the name of Lyens, a resident of Market street. This man, who is about sixty-three years of age, and who is married and the father of a numerous family, was prosecuted, a short time ago, in our County Court, on account of rape and bastardy, alleged to have been committed upon an Irish female, a widow, formerly in his employ as a house-servant. At the time of the trial the child was two months old. A number of witnesses, professional and non-professional, were subpœnaed on both sides. For the proof of the bastardy, the testimony of the mother of the child was exclusively relied upon. She stated that some time in the month of March last the defendant had knocked her down, and that, while she was in a state of partial unconsciousness, he had ravished her person, conception being the consequence. Unfortunately, however, for the validity of her testimony, it was proved that she was delivered, precisely eight months after the occurrence, of a full grown child. Her testimony, moreover, was in many other particulars extremely loose and contradictory. On the part of the defendant, it was shown that he had been affected, for several years, with a large scrotal hernia, which had incapacitated him for sexual intercourse, by retracting the verile member, and preventing successful emissions. The tumor, according to my admeasurements, was twenty inches in circumference, and nine inches in length when the man was recumbent, and nearly twelve inches when he was in the erect posture. Occupying the right side, it was irregularly pyriform, and exquisitely tender on pressure. The testicle was situated on the lateral aspect of the tumor, near its middle, and almost directly opposite the left. The entire penis, with the exception of the head, was concealed by the rupture, and dragged out of its natural position. The contents of the tumor were irreducible. The man was stout and well-built, with a decided inclination to corpulency.

Several intelligent medical gentlemen were called upon the stand, and their testimony tended to show that, while intercourse was possible, it was not, under the circumstances, at all probable. One or two even went so far as to declare that they considered it almost physically impracticable for a man, situated like the defendant, to effect penetration, their opinion being based upon the concealed and crooked condition of the verile member, the large volume and tender state of the tumor, and the prominence of the abdomen, which were regarded by them as so many circumstances calculated to interfere with the consummation of the act. Those who admitted the possibility of the act agreed with the other witnesses that it could have taken place only while the woman was in the most passive condition ; or, in other words, that the slightest resistance, on her part, would have been sufficient to enable her to preserve her chastity, had she been so inclined. One of the medical witnesses testified that the size and age of the tumor were such as to weaken the seminal organs, to destroy sexual desire, and to render any successful emissions extremely difficult, if not impracticable. The presiding Judge, the Hon. Mr. Garland, charged the jury in consonance with the declaration of the medical men, and the fact that the child, although full-grown, had been born at the expiration of eight months from the time of the alleged intercourse, and they accordingly found the man "not guilty."

The effect of large scrotal herniæ in relation to impotence has not, I think, sufficiently claimed the attention of medical jurists. Most writers upon the subject give it a mere passing notice, without adducing any satisfactory information in illustration of it. Fodere, in his learned "*Traité de Médicine Légale et d'Hy-*

*giène Publique*," published in 1796, makes a distinct recognition of the circumstance, declaring that a scrotal rupture of large size might, by its continued pressure upon the spermatic vessels, prevent the secretion of the seminal fluid. In Italy, he states, double hernia, by pressing on the spermatic cords, sometimes causes as complete emasculation as when the testicles are removed with the knife. Many of the fine singers of that country are persons laboring under this disease, having been rendered so by the visitation of God.* METZGER, a writer on medical jurisprudence, informs us that the Medical College of Western Prussia declared the existence of a large and irreducible hernia a sufficient cause of divorce. The late Professor ORFILLA† observes that a scrotal rupture may be so voluminous as almost to efface the penis, and thus render coition impracticable. The same effect, he thinks, may be produced by a very large hydrocele. Our distinguished countryman, Dr. T. R. BECK, the able author of the "Elements of Medical Jurisprudence," the most erudite work on the subject in modern times, admits the possibility of such an occurrence with regard to scrotal hernia, principally, it would seem, upon the authority of FODERE; and extraordinary brevity of the penis, however induced, is generally considered by medico-legal writers and teachers as a disqualifying circumstance.

Three things are indispensably necessary in the male to successful copulation—"*erectio ac intromissio penis, cum emissione seminis.*" In the case of Lyens it is doubtful, from the volume of the tumor, the great brevity and crookedness of the verile member, and various other circumstances, whether he could command the one, or perform the other. As the hernia, however, is single, it is likely that he may have an occasional emission; but whether such a discharge could take place at a particular moment, under the influence of sexual excitement, is extremely problematical. In their attempt to arrive at a correct decision upon the subject, the jury were probably not altogether governed by the condition of the defendant, but, in some degree, by the character of the plaintiff, which was, to say the least, more than suspicious.

Finally, a large scrotal hernia might, juridically speaking, be-

* Op. cit T. 1. p. 373.

† Leçons de Medicine Legale, T. 1. p. 145. Paris, 1828.

come a source of interest in another point of view. A man thus affected might, in a rencountre with another, be struck or kicked upon the tumor, and thus sustain fatal injury, either from the shock of the blow, or from a rupture of the bowel, and consequent extravasion of fœcal matter. In the latter case, he might, it is true, escape with an artificial anus; but the greater probability, by far, would be that he would die from peritoneal inflammation. It would be a nice question, in such a case, for a medical jurist to determine whether the criminal should be punished with the same severity as when life is destroyed where no such complication exists. The sophist would have no difficulty in finding a satisfactory solution.

---

### Case 2.—*Encephaloid of the Mammary Gland.*

The patient, Miss H., is a resident of Barren county, Kentucky, and is thirty years of age. She came to me, a few days ago, as a private patient, with a letter of introduction from my friends, Drs. Jourdon & Trabue, of Glasgow, under whose treatment she had been a short time previously. She is the daughter of a poor farmer, has always been industrious in her habits, and, until attacked with her present disease, was never sick. Upon inquiry, she informs me that one of her grand-mothers was reported to have had cancer of the breast, but that she was cured of it, and lived thirty years afterwards, when she died of another complaint. It would seem that this female, about ten years ago, received an injury upon her right breast, producing slight inflammation, which, however, soon disappeared, but left a small movable nodule about two inches to the left of the nipple. The little tumor gradually but slowly increased, and was occasionally the seat of sharp, lancinating pains. About the middle of June, 1853, it had attained the size of a large walnut. It now began to grow very rapidly, and became the seat of a burning pain, more severe at one time than at another. The skin retained its natural color until the middle of last August, when it began to assume a purplish aspect at the part where the morbid growth was first observed. About two weeks before her arrival in town, a physician, supposing there might be pus in the tumor, punctured it at two points. Nothing escaped from the lower opening, but the upper discharged a large quantity of fluid which, at first, seemed to be pure blood, but which soon changed to a whitish, flaky, and offensive matter. Within a short time after the swelling was punctured, a fungus commenced protruding at each orifice, and is now, owing to the destruction of the intervening integuments, of enormous size, of a dark purple color, and the seat of frequent and copious hemorrhages. The pain, since the formation of the fungus, has become much mitigated. At present, the tumor measures twenty-two inches in circumference, and twelve inches across its surface. The subcutaneous veins are exceedingly enlarged, much more so than I have witnessed in any other case, and they are seen ramifying over the tumor like blackish lines, the blood in several of them being seemingly completely stagnant. Altogether the appearance of these vessels is most singular and striking. The tumor, which is somewhat hemispherical in shape, is elastic on pressure, evidently lobulated, and pretty firmly adherent to the pectoral muscle. What is remarkable, it is the seat of very little pain. The discharge from the enormous ulcer is very profuse, thin, sanious, and excessively fœtid. There is no enlargement of the glands of the axilla, of the subclavicular region, or of any other part of the chest.

The general health, as you observe, is much deranged; the countenance is pale and sallow, the muscles are flabby and attenuated, the pulse is habitually feeble and above a hundred in the minute, the bowels are constipated, and her strength is rapidly declining. For the last fortnight she has had occasional paroxysms of fever, attended with chilly sensations, but no sweats. Her sleep is much interrupted, and she is obliged to use anodynes at night. Her appetite is feeble. It may be stated that there is occasionally a keen, darting pain in the other breast.

Such, gentlemen, is a faithful outline of the case before us, one, in every respect, of the most melancholy character, from the fate which inevitably awaits it. Surgery, in such a disease, and especially at such a stage of it, is vain and impotent, utterly without hope and without resources. Our hands are literally tied; the time for an operation, if an operation is ever justifiable in such a disease, is passed; and all that we can do, under the circumstances, resolves itself into mere attempts at palliation. The great and leading indications are, first, to support the woman's strength; secondly, to allay the excessive fœtor of the discharges; and, thirdly, to prevent further hemorrhage, the continuance of which must speedily destroy her. Already the very foundations of life have been sapped by the frequent and abundant losses which she has thus sustained.

To meet the first indication, I shall order her to take, every five hours, or four times daily, at equal intervals, two grains of quinine and the same quantity of sulphate of iron, in solution, along with twenty drops of elixir of vitriol. The exhibition of these articles will, if anything can, under the circumstances, improve the tone of the digestive organs, increase the strength, and impart a certain degree of plasticity to the blood, which, as is apparent from the countenance, is in a highly impoverished condition. The diet should be plain but as nutritious as possible, and should consist, mainly, of animal broths, with the addition of soft-boiled rice, barley, or hominy, the lighter kinds of meats, and stale bread, crackers, or soda biscuit, with weak tea or milk at breakfast and supper. Coffee, hot bread and biscuit, pastry, and the coarser varieties of meats and vegetables should be carefully avoided. Porter, good ale, wine-whey, or milk-punch, should be taken several times a day, in quantities suited to the state of the stomach and heart. There is no probability that any of these articles will injuriously excite the circulation, and I shall, therefore, recommend them to be used quite freely. To induce sleep, and allay pain, anodynes should be given, but not oftener or in larger quantity than is absolutely necessary to attain these objects.

The discharges being excessively fœtid, it is necessary to pay some attention to this circumstance, both on account of the patient, and for the sake of those who are obliged to be around her. You may easily suppose what would be the effect upon a system, already so enervated as this poor patient's, if she were compelled to breathe an atmosphere so greatly contaminated by the effluvia of her own person. Its tendency would inevitably be to produce still further depression of the vital powers by impairing her appetite and preventing the due æration of the blood. We shall, therefore, direct, not merely some, but special attention to be paid to this subject. The remedy by which we may hope to allay this horribly offensive and sickening odor is the liquid chlorinate of soda, a drachm of which may be mixed with about four ounces of water, which may then be sprinkled, from time to time, upon the dressings, and also upon the body and bedclothes, or a piece of lint, wet with it, may be kept constantly upon the ulcerated surface. The liquid chlorinate of lime may also be used, but I usually prefer the soda. It need hardly be stated that the dressings should be frequently changed, and the parts cleansed with tepid water.

The third indication is not so easily fulfilled; for the fungus will be likely to bleed, more or less, in spite of all that can be done to prevent it. The internal remedies, if faithfully employed, may do something towards this, by increasing the plastic powers of the blood; but I do not think that we need look for much aid from this quarter. My chief reliance will be upon rest and suspension of the affected organ, and upon the application of creosote, alum, and acetate of lead, at the commencement of a threatened attack of hemorrhage. As a preventive, care should be taken to avoid all compression of the tumor by bandages and other means. When the blood spirts out, as it occasionally does, in a full stream, from an enlarged vein or artery, direct compression with the finger will prove the most efficient remedy, and I shall not fail to give the patient some instruction upon this point.

Whether the above measures will be instrumental in promoting the comfort of our patient, and in prolonging her existence, cannot be positively predicted. Everything indicates that she cannot, under any circumstances, survive beyond a few weeks; but it is our duty to do something, and the above course is the best that suggests itself to my mind.

Case 3.—*Scirrhous of the Mammary Gland.*

Mrs. Vie, aged thirty-two years, a native of Germany, but for the last eight years a resident of this country, and the mother of six children, the youngest being sixteen months old, noticed, about four years ago, immediately on the inner side of the left nipple, a small tumor which has been steadily but tardily increasing ever since, and is now about the volume of a very large orange. It is lobulated on the surface, or, more correctly speaking, it looks as if it were composed of several small masses, free at the top, but fused together at their base, remarkably hard, and but slightly movable. The skin is somewhat rough, of a tawny, reddish color, and pervaded by numerous little vessels, in a state of considerable turgescence. At one point, over a space about half an inch in diameter, it is superficially ulcerated. It was for several years entirely free from uneasiness; but within the last few months, it has been the seat of occasional pain, of a darting, pricking, and burning character, and particularly severe in damp states of the atmosphere. The axillary ganglions are slightly enlarged and exceedingly hard, though quite movable, and a similar nodule exists along the inferior edge of the great pectoral muscle and also just below the clavicle. The nipple retains its normal shape and size, but is somewhat more firm than naturally. Such is a brief but accurate account of the condition of the breast and of the surrounding parts. The general health, you will notice, is but little impaired. Latterly, however, the woman has lost some flesh, and her strength is not as good as formerly, though she is still able to attend to her house-work. She menstruates regularly; her appetite is natural, and she sleeps well enough at night. The bowels are habitually costive, and the spirits are somewhat depressed. So far as I can learn, there is no hereditary predisposition to carcinomatous disease. The eyes and complexion are light, and the stature rather diminutive.

There are several points of interest in this case demanding brief notice. In the first place, our attention is arrested by the patient's age. She is now thirty-two years old, and the malady, as we are informed by the record, was first noticed four years ago. It is quite unusual to witness scirrhus of the breast at this early period. In the great majority of cases it does not show itself before the age of forty, forty-five, or fifty; that is, about the decline, or soon after the disappearance, of the menses. Indeed, there are some writers who deny that genuine scirrhus is ever seen in this gland under the age of thirty-five, or forty; but this is a mistake, as my own experience testifies. Instances of this disease occasionally occur, though very rarely, in females between the ages of eighteen and twenty-five; and there are several cases on record where it was witnessed before the period of puberty.

Secondly, we may mention, as singular features, the small volume of the tumor, its excessive hardness, and the peculiar tubercular appearance of its surface. Scirrhous formations never acquire any great bulk; but in this instance the growth is uncommonly small, especially when we consider its age. The consistence is also unusual. I do not recollect ever to have felt so hard a mammary gland from any cause. The tumor projects off from the surface of the chest, and its surface has a singularly tuberculated appearance. What adds to this appearance is the want of adipous matter which isolates the indurated and enlarged gland from the circumjacent parts, and thus renders it inordinately prominent.

In the third place, there is a peculiarity in the nipple in this case. In most instances of scirrhous of the mammary gland, this little body is greatly retracted, especially in the advanced stages of the complaint, as well as singularly altered in its form and appearance. In the present case, on the contrary, it retains its normal characters, with the exception, perhaps, of its being a little harder than in the natural state.

Fourthly, it is not common to witness such extraordinary hardness in the lymphatic ganglions of the axilla, on the pectoral muscle, and in the subclavicular region. The sensation which these bodies impart to the finger leads to the conviction that they are deeply involved in the morbid action; or, to express myself more properly, that they are in the same scirrhous condition as the mammary gland itself.

Fifthly, although the disease is of four years standing, there is, as yet, comparatively little derangement of the general health. All the functions, even the menstrual, appear to be well performed. The malady, however, is gradually progressing, and will, doubtless, ere long, make serious inroads upon the general system.

Having thus explained to you the chief points of interest in this case, let me say a few words respecting its diagnosis. I have repeatedly stated to you, in the course of the preceding remarks, that the disease is scirrhous, or hard cancer, and you will necessarily expect that I should assign some reason for my opinion. Any elaborate comments upon the subject, however, would, after what has been already said, be superfluous. The symptoms are so plainly marked, so distinct and characteristic, that he who runs may read. The slow but steady progress of the tumor, its excessive hardness, which is greater than that of any other substance, except cartilage and bone, its small volume, its circumscribed form, its tuberculated surface, the sharp, pricking pain, and the enlarged and indurated condition of the neighboring lymphatic ganglions, are so many circumstances which stamp the nature of the disease, and enable us, even upon the most superficial examination, to determine the diagnosis with unerring certainty. In short, there is no other tumor, deposit, or formation, of which I have any knowledge, or which occurs in the mammary gland, which at all resembles the one before us.

A month ago, I showed you a case of encephaloid tumor of the mammary gland, and, if you will recall to mind the circumstances attending it, you will be struck with the remarkable contrast between that case and this. In the former, the tumor was of enormous volume, most of which it had attained in a very few months; it was comparatively soft and elastic; there was excessive enlargement of the subcutaneous veins; the centre of the morbid mass was ulcerated, and was the seat of a foul, sanious discharge, together with frequent hemorrhages; the pain was so great as to compel the poor woman to use anodynes to enable her to sleep; the neighboring lymphatic ganglions however, even those of the axilla, were free from disease; the body was greatly emaciated; and the countenance exhibited a peculiar sallow and cachectic appearance, indicative of a contaminated state of the system, and of approaching dissolution. No two cases could possibly be more dissimilar, or ex hibit, in a more striking manner, their distinctive peculiarities.

A few words in regard to the treatment of this case, and we shall dismiss it. The patient has applied to me for relief. She has borne up nobly under her burden, but it is becoming more and more painful, and she is, therefore, anxious to have something done. It must be obvious to you from the account of the case, and from the general remarks which I made to you upon carcinomatous affections in the early part of the session, that the case is not a proper one for the knife. Such is the condition of the tumor and of the neighboring parts that the removal of the diseased gland would only tend to hasten the poor woman's death. Nay, it admits of doubt, great doubt, whether a scirrhous formation of the mammary gland should ever be treated in this manner, even under the most favorable circumstances, as it respects the surrounding structures and the general health. My opinion has always been—and this is the doctrine which I have endeavored to instil into the minds of my pupils ever since I began to teach the principles and practice of surgery—that the less we meddle with these cases the better it is for our patients; the less likely, in short, will they be to have their sufferings aggravated and their lives prematurely destroyed. For a number of years past I have declined operative interference nearly altogether, even in the earliest stages of the disease, and in the report which I had the honor recently to prepare for the American Medical Association, "On the Results of Surgical Operations in Malignant Diseases," I have endeavored to place this subject in its true light before the profession. The facts developed in that document are of a most startling character, and cannot fail, I think, to satisfy the most sceptical in regard to the propriety of the rules there laid down for our government in the treatment of malignant affections. I trust that the reading of that paper, if you should so far honor it, and my oral teaching in this institution, will have a tendency, on the one hand, to inspire you with a love for conservative surgery, and, on the other, to repress any itching that you may have for the indiscriminate use of the knife.

But to return from this degression. Since the knife is inadmissible in this case, we must content ourselves with palliatives; that is, means calculated, as in the case of Miss H., to improve the general health, and to allay pain. With this view we shall order the patient to take occasionally a few blue pills, to regulate the bowels and secretions; to live upon light and simple food; and to use, three times a day, at equal intervals, two grains of citrate of iron and sulphate of quinine, with a little morphia, laudanum, or Dover's powder at night. As to arsenic and cicuta, two articles which have acquired some celebrity in the treatment of carcinomatous diseases, I have, after repeated trials, found them utterly worthless, and now never employ them. The dress should be worn very loose, so as not to compress the tumor, which should be kept constantly covered with a layer of glazed cotton; a substance that will afford sufficient warmth, and at the same time maintain the cutaneous perspiration.

